# Radiological evaluation of malignant pleural mesothelioma - defining distant metastatic disease

**DOI:** 10.1186/s12885-020-07662-y

**Published:** 2020-12-09

**Authors:** Dearbhaile Catherine Collins, Raghav Sundar, Anastasia Constantidinou, David Dolling, Timothy Anthony Yap, Sanjay Popat, Mary E. O’Brien, Udai Banerji, Johann Sebastian de Bono, Juanita Suzanne Lopez, Nina Tunariu, Anna Minchom

**Affiliations:** 1 Drug Development Unit, Royal Marsden Hospital/ Institute of Cancer Research, Down Rd, Sutton, SM2 5PT UK; 2 Lung Unit, Royal Marsden Hospital, Fulham Rd, London, SW3 6JJ UK; 3 Lung Unit, Royal Marsden Hospital, Sutton, SM2 5PT UK

**Keywords:** Mesothelioma, Metastases, Pleural mesothelioma, Bone metastases

## Abstract

**Background:**

Malignant pleural mesothelioma (MPM) is traditionally characterized by local destructive spread of the pleura and surrounding tissues. Patient outcomes in MPM with distant metastatic dissemination are lacking.

**Methods:**

In this retrospective study, we reviewed a cohort of 164 MPM patients referred to a Phase I trials unit, aiming to describe identified metastatic sites, and correlate with clinical outcomes.

**Results:**

67% of patients were diagnosed with distant metastatic disease with a high incidence of bone (19%), visceral (14%), contralateral lung (35%) and peritoneal metastases (22%). Peritoneal metastases were more likely in epithelioid versus biphasic/ sarcomatoid MPM (*p* = 0.015). Overall survival was 23.8 months with no statistical difference in survival between those with distant metastases and those without.

**Conclusions:**

This report highlights the frequency of distant metastases and encourages further radiological investigations in the presence of symptoms. In particular, given the relatively high incidence of bone metastases, bone imaging should be considered in advanced MPM clinical workflow and trial protocols. The presence of distant metastases does not appear to have prognostic implications under existing treatment paradigms. This cohort of MPM patients gives an indication of patterns of metastatic spread that are likely to become prevalent as prognosis improves with emerging treatment paradigms.

## Background

Malignant pleural mesothelioma (MPM) is a cancer of the pleura characterized by local spread and destructive infiltration. It is strongly associated with exposure to asbestos fibres with a lag time of thirty to forty years prior to development and diagnosis. MPMs are divided according their histopathological appearances, with epithelioid, comprised of polygonal, oval, or cuboidal cells, the predominant subgroup. Less common histopathological variants include sarcomatoid MPMs, consisting of spindle cells, and the biphasic subtype containing both epithelioid and sarcomatoid areas within the same tumour.

MPM has been historically described as having a local pattern of disease spread [1–3]. In recent years, evidence for alternate or unexpected patterns of metastatic spread in MPM have largely been from case reports or autopsy series. A case series from postmortem examinations of 318 patients has quantified rates of liver metastases of 32%, splenic metastases of 11%, thyroid metastases of 7% and brain metastases of 3% [4]. A further systematic review of data from autopsy studies reported an incidence of brain metastases of 2.7% [5]. Others report nodal metastases in 40% of patients [6]. Although imaging techniques and availability of specialist radiology has improved, there still remains a paucity of documentation of the patterns of MPM metastatic spread.

Standard systemic anticancer treatment for MPM includes chemotherapy, usually a platinum compound in addition to the folate antimetabolite, pemetrexed [7–9]. Other chemotherapy options include vinorelbine although response rates in the second line setting and onwards are poor. Immunotherapy has shown promise in early phase trials, though later phase trials are not yet fully reported [10]. Therefore, many MPM patients are referred to clinical trial units for novel investigational compounds. In general, early phase trial patients are fitter, with superior performance status than the general cancer population due to rigorous trial eligibility requirements. These include almost perfect performance status in addition to normal haematological and biochemical parameters and underlying organ function. Performance status has been repeatedly shown in studies to be an independent predictor of overall survival and better prognosis [11–13]. Thus, this trial population offers a unique subgroup of MPM patients who may live beyond the expected median survival and undergo extensive diagnostic imaging as part of trial enrollment, presenting an opportunity to document distant metastatic spread in MPM.

We set out to review our MPM cohort of patients referred for early phase clinical trials at the Drug Development Unit (Royal Marsden Hospital/ Institute of Cancer Research). We aimed to document the location of clinically and radiologically identified metastatic disease thus determine the incidence and pattern of metastatic spread from mesothelioma and correlate with clinical outcomes.

## Methods

### Patient demographics

This was a retrospective review of MPM patients from a prospectively maintained database. Approval was obtained from the local research and audit committee. All MPM patients who attended the Drug Development Unit at the Royal Marsden Hospital and Institute of Cancer Research, London, UK, were included for analysis, regardless of whether they were subsequently enrolled upon a clinical trial. Demographic, clinical, pathological, and radiological data were gathered for MPM patients referred to the Drug Development Unit from January 1992 to January 2017 with follow up data until January 2018. Data collected included all prior treatments and tumour response to these therapies in addition to time to tumour progression. Early phase trial information including the investigational compound and specific trial enrolled upon was collected as well as tumour response rates. Survival data where known or last date of clinical review were collected for overall survival analysis.

### Identification of metastases

All available radiological tests and imaging platforms were included in the analysis in order to identify sites of metastatic disease. This included plain film radiography (X-ray), computed tomography (CT), ultrasound (US), isotope bone scans, magnetic resonance imaging (MRI), echocardiography and positive electron tomography (PET). Imaging was comprehensively reviewed by a single, dedicated early phase trial radiologist to verify sites of metastatic disease for trial eligibility and response assessments. As the majority of patients were referred from an external hospital, only their external scans at time of referral were submitted and were available for review. All imaging performed at the Royal Marsden Hospital was reviewed. In addition to radiological reports, clinical notes and multidisciplinary meeting outcomes were reviewed extensively and documented clinical sites of metastasis included, such as cutaneous and subcutaneous lesions and drain site metastasis. Where data were missing or incomplete, these were excluded from final survival analysis.

### Statistical analysis

Descriptive statistics were used to summarise patient, past treatment and tumour characteristics including the various sites of metastatic spread identified through radiological review. Overall survival calculated in months from date of diagnosis of MPM to date of death. Overall survival was determined using Kaplan-Meier analysis presented as overall survival (OS) and hazard ratios (HRs) were estimated using univariate Cox regression models. Data were presented as survival plots. All tests were 2-sided, and *P* ≤ 0.05 was considered to indicate statistical significance. Descriptive statistics and survival analyses were performed using Stata, version 13.1. Other analyses used Graphpad Prism, version 7.

## Results

### Baseline characteristics

One hundred sixty-four patients referred for consideration of enrollment into early phase clinical trials that were evaluable for metastatic disease sites and survival over the time period. They were predominantly male (76%) with a median age at MPM diagnosis of 64 years (Table 1). Available histopathology from reports and referral letters was reviewed in all cases. The subtype of mesothelioma was identifiable in 84% of cases (*n* = 138) and was mostly epithelioid (*n* = 115, 83%), with 9% biphasic (*n* = 12) and 8% sarcomatoid type (*n* = 11) (Table 1).

**Table 1:** Demographics and histopathological diagnosis

Demographics	N (%)
Gender	
Male	125 (76%)
Female	39 (24%)
Median age at diagnosis	
64 years (range 37–90)	
Histopathology	
Epithelioid	111 (68%)
Biphasic	12 (7%)
Sarcomatoid	11 (7%)
Other	8 (5%)
Unknown subtype	22 (13%)

MPM patients had a median of three imaging modalities reviewed for assessment of metastatic disease ranging from one to five. This was predominantly comprised of CT scans, of which patients had a median of two scans reviewed (range 1–9), and plain radiographs, of which patients had a median of 1 (range 0–6) and this was almost exclusively a chest radiograph.

Patients who were referred for early phase clinical trials had received a median of two prior chemotherapies, ranging from none to six (Table 2). Five patients were referred directly to the early phase trials unit prior to conventional first line therapies. Of the remaining 159 patients, a platinum-based chemotherapy regimen with pemetrexed was initiated in 71% of cases (*n* = 117) in the first line setting. The remaining patients received alternatives to pemetrexed such as vinorelbine (6, 4%), clinical trial enrolment (21, 13%) or MVP (mitomycin-C, vinblastine and cisplatin) (17, 10%). The majority of patients who presented to the early phase trial unit had undergone a second regimen of anticancer treatment (117, 71%) with half referred for clinical trials for their second line of treatment (58, 50%). Of the remaining 59 patients, almost one quarter were rechallenged with a platinum and pemetrexed (27, 23%). The remainder underwent a variety of second line chemotherapies, most frequent of which was vinorelbine (17, 15%), but also ifosfamide, gemcitabine, MVP and TACE (mitomycin-C, gemcitabine and cisplatin).

**Table 2:** Prior treatments and interventions

Prior treatments / interventions	N (%)
Surgery / intervention	
Thoracoscopy +/− intervention	53 (32%)
No surgery / intervention	37 (23%)
Cardiothoracic surgery	31 (19%)
Other intervention	11 (7%)
Unknown surgery / intervention	32 (20%)
First line anticancer treatment (*n* = 164)	
Platinum + pemetrexed	117 (71%)
Clinical trial	21 (13%)
MVP (mitomycin C, vinblastine, cisplatin)	15 (9%)
Other chemotherapy regimen	6 (4%)
Incomplete / missing information	5 (3%)
Second line anticancer treatment (*n* = 117, 71%)	
Clinical trial	58 (50%)
Rechallenge platinum and pemetrexed	27 (23%)
Other chemotherapy regimen	26 (22%)
Incomplete / missing information	6 (5%)
Median total treatments prior to referral	2 (range 0–6)

### Site of primary MPM and metastases

Of all MPM included for analysis, 64% (*n* = 106) were right sided with the remaining MPM localised to the left-hand side (*n* = 58, 36%).

The spread of metastatic disease was divided into local and distant sites and are outlined in Table 3. Local spread included pleural effusion(s) documented along the course of the MPM disease in 102 patients (62%). Other local sites included mediastinal and paratracheal lymphadenopathy that was radiologically considered and reported as “pathological” in 101 patients (62%). Chest wall involvement was noted in 68 patients (42%). The pericardium was considered infiltrated in 47 patients (29%) with evidence of a pericardial effusion in 19 of these patients (12% total).

**Table 3:** Site of metastatic disease

	Location	N (%)
Local disease spread	Pleural effusion	102 (62%)
	Thoracic nodal disease	101 (62%)
	Chest wall involvement	68 (42%)
	Pericardial infiltration	47 (29%)
	Pericardial effusion	19 (21%)
Distant metastatic sites (*n* = 110, 67%)	Contralateral lung disease	55 (35%)
	Parenchymal lung metastasis	42 (26%)
	Peritoneal / omental metastasis	36 (22%)
	Ascites	24 (15%)
	Bone metastasis	31 (19%)
	Visceral metastasis	23 (14%)
	Liver	19 (70%)
	Renal	3 (11%)
	Adrenal	3 (11%)
	Spleen	2 (8%)
	Brain metastasis	5 (3%)
	Subcutaneous metastatic nodules	32 (20%)
	Intramuscular metastasis	7 (4%)

Distant sites of disease are also recorded in Table 3. A total of 110 patients (67%) had at least one documented site of distant metastasis with many patients having multiple distant sites diagnosed synchronously or metachronously. They include contralateral lung disease noted in 55 patients (35%) and multiple parenchymal lung metastasis in 42 (26%). In 17 cases which equated to 10% of the total patient cohort and 31% of patients with parenchymal lung metastasis, radiographic appearances were of a diffuse military-type metastatic pattern. Peritoneal disease and/or omental involvement was noted in 36 patients (22%), with ascites evident in 24 patients.

**Fig. 1:**
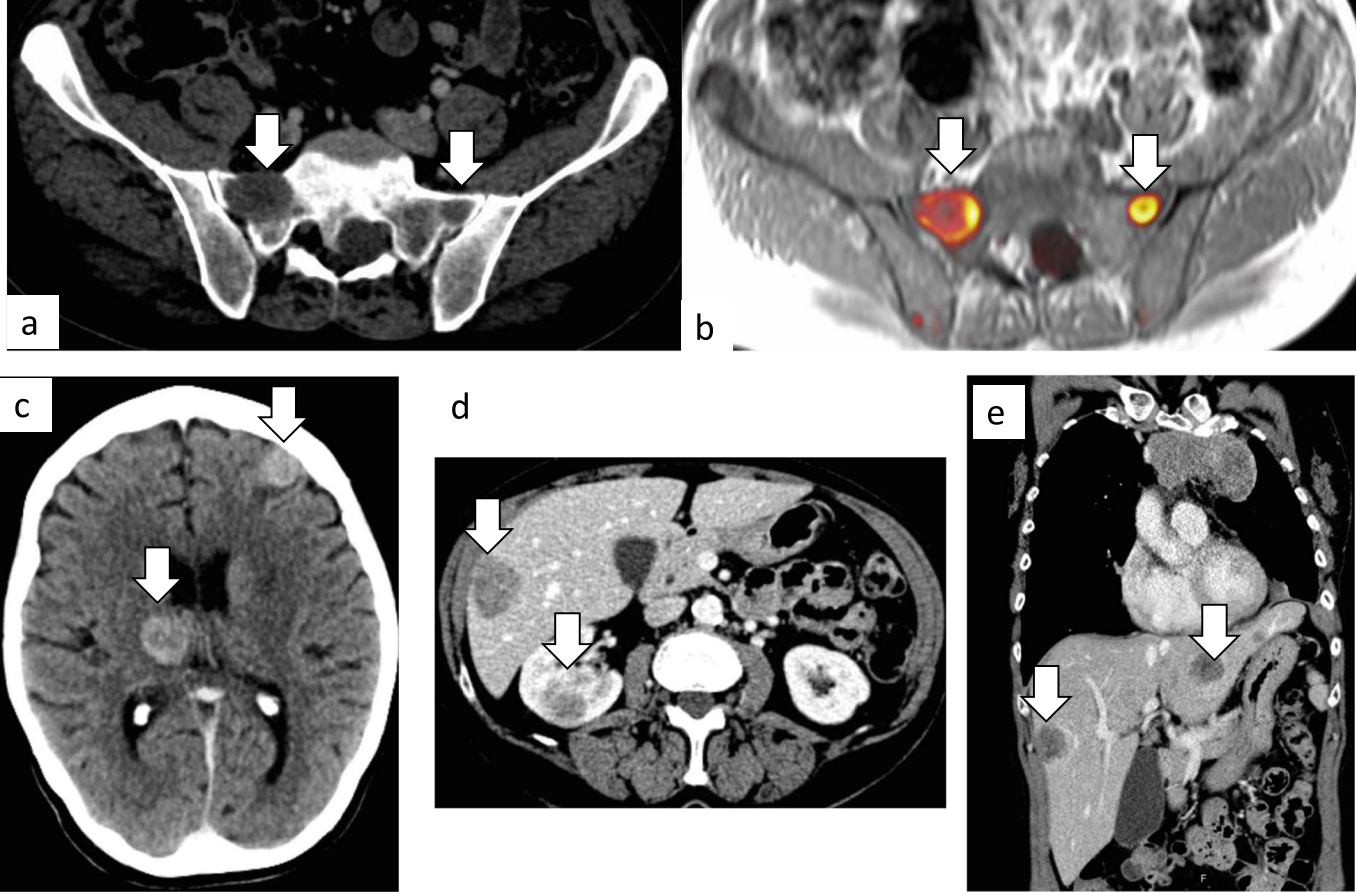
Radiological imaging of distant metastatic sites. Axial contrast enhanced CT (**a**) and fused DWI-T1W-MRI (**b**) images showing lytic bone metastases in the sacrum bilaterally. Axial contrast enhanced CT showing brain metastases (**c**). Axial enhanced CT (**d**) showing liver and renal metastases. Coronal enhanced CT (**e**) showing liver metastases. *CT: computer tomography; DW1: diffusion weighted 1, T1W: T1 weighted, MRI: magnetic resonance imaging*

Bone metastases were diagnosed in 31 patients (19%) (Table 3). Of the bone lesions, the majority (18, 60%) were documented as “lytic” on radiological imaging, with 7 (23%) defined as “sclerotic” and 5 (17%) unknown or unremarked upon. Visceral metastases included distant spread to the major intra-abdominal organs including the liver, kidney, adrenal and spleen. Twenty-seven sites of disease were documented in 23 patients (14% of total). Of these visceral lesions, most were located in the liver (19, 70%), three in the kidney, three in the adrenal gland(s) and two patients (8%) had radiologically diagnosed splenic metastases. Only five patients (3%) had brain metastases confirmed radiologically. All these patients had neurological symptoms that prompted imaging. Subcutaneous metastatic nodules distant to the site of MPM and distant to any potential chest drain or biopsy site was noted in 20% (*n* = 32). Seven patients (4%) had distant intramuscular metastasis including gluteal muscle, psoas muscle and distant intercostal muscle deposits. Figure 1 depicts radiological images of distant metastatic sites. On comparison of distribution of sites of metastases by histological subtype, peritoneal metastases were more likely to occur in epithelioid compared to biphasic/ sarcomatoid MPM (frequency of 27.0% in epithelioid and 4.3% in biphasic/ sarcomatoid; *p* = 0.015). Though there were higher rates of bone metastases in biphasic/ sarcomatoid compared to epithelioid subtypes, this did not reach statistical significance (frequency of 18.2% in epithelioid and 34.7% in biphasic/ sarcomatoid; *p* = 0.0936) (Table 4).

**Table 4:** Metastatic spread according to histopathological subtype. *Subcut: subcutaneous, Mets: metastases*

Pathology	N	Sites of Metastases						
		Effusion	Contralateral lung mets	Bone mets	Visceral mets	Brain mets	Peritoneal	Subcut/ muscular
Epithelioid	115	74 (64.3%)	38 (33.0%)	21 (18.2%)	16 (13.9%)	3 (2.6%)	31 (27.0%)	23 (20.0%)
Biphasic/ Sarcomatoid	23	12 (52.1%)	9 (34.7%)	8 (34.7%)	4 (17.4%)	2 (8.6%)	1 (4.3%)	5 (21.7%)
Comparison of proportions (Fishers exact test)		*P* = 0.3464	*P* = 0.6277	*P* = 0.0936	*P* = 0.7455	*P* = 0.1938	*P* = 0.0159	*P* = 0.7839

### Patient survival

**Fig. 2:**
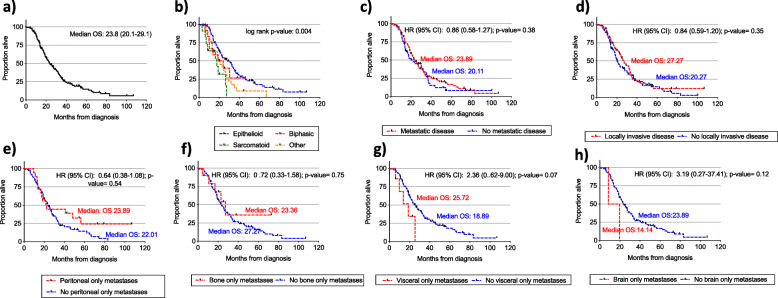
Median overall survival (months) of entire MPM population referred for consideration of early phase clinical trials (**a**). Median overall survival (months) according to histopathological subtype (**b**). Median overall survival (months) according to presence of metastatic disease (**c**) or presence of locally invasive disease (**d**). In those with metastatic disease median overall survival (months) according to presence or absence of peritoneal only metastasis (**e**), bone only metastasis, (**f**) visceral only metastasis (**f**), and brain only metastasis (**g**). *OS: overall survival*

In this dataset, total overall median survival of patients in our dataset was 23.8 months (range 1 month to 106 months) from initial diagnosis of MPM to death (Fig. 2a). The precise date of death was known in 122 patients (74% of total cohort), the remainder were discharged from the Drug Development Unit to their primary oncology unit and outcome was unknown. Epithelioid histological subtypes had a statistically significant better overall survival than biphasic and sarcomatoid subtypes (*p* = 0.004) (Fig. 2b).

The presence or absence of metatatic disease or locally invasive disease did not correlate with overall survival (Fig. 2c and d). In those with metastatic disease, those with peritoneal or omental only metastases had no difference in overall survival compared to those without (23.9 vs 22.0 months, HR 0.64, *p* = 0.54). There was a difference of 4 months survival with the presence or absence of bone only disease alone though this did not reach statistical significance (23.4 months vs 27.3 months, HR 0.72, *p* = 0.75) (Fig. 2e). There was a difference of just under 7 months survival with the presence or absence of visceral only metastasis though not reaching statistical significance (18.9 months vs 25.7 months, HR 2.36, *p* = 0.07) (Fig. 2g). The presence of brain metastases had a poor median overall survival of 14.1 months compared to a median survival of 23.9 months though the comparison was limited by low numbers of patients with brain only metastases (HR 3.19, *p* = 0.12) (Fig. 2h).

### Early phase clinical trial details

Of the 164 patients who were referred for consideration of early phase clinical trials, 92 (56%) were identified as suitable for allocation onto a trial with 60 (37%) deemed ineligible for enrollment and the remaining 12 (7%) were unknown whether they were deemed eligible for enrolment upon a clinical trial. Of the 92 patients who went forward for clinical trial screening, 69 went commenced on an early phase trial (75%) and 23 did not ultimately embark upon a study due to trial ineligibility (10, 43%), patient deterioration (8, 35%), or patient decision (5, 22%). Of those that received an investigational compound, the predominant radiological tumour response was progressive disease in the majority (39, 57%), with 25 (36%) having stable disease and one confirmed complete response and one confirmed partial response (1% respectively). Three patients (4%) were non-evaluable due to discontinuing the trial prematurely and not being radiological assessable.

## Discussion

We aimed to describe the local and distant metastatic patterns of advanced MPM patients referred to our dedicated early phase clinical trial unit and correlate with clinical outcome. Previous evidence for the pattern of metastatic spread in mesothelioma has been largely derived from small cohorts or autopsy series, reporting liver metastases in around 30% and nodal metastases in 40% [4–6]. However, our study had the advantage of including advanced MPM patients who received multiple and frequent imaging modalities as part of early phase clinical trial enrolment, in excess of what might be arranged as part of routine clinical follow up.

In this analysis, 67% were diagnosed with metastatic lesions at a variety of distant locations. While brain metastases remained uncommon at 3%, an unexpectedly high incidence of bone (19%) and visceral metastasis (14%) were recorded. There were variations in pattern of metastatic spread between histological subtypes with peritoneal metastases more common in epithelioid subtypes. The bone lesions were predominantly lytic in nature. To the best of our knowledge, the incidence of bone metastases has not been reported before in MPM. This is likely because previous evidence has been from case reports and autopsy series (in which bones were not routinely examined with imaging) whereas our series uses detailed radiology, predominantly CT imaging and plain X-rays. Detailed knowledge of the potential sites for MPM spread is essential for proactive investigation and management. There was no correlation found between presence of bone metastases and overall survival in this series but bone fractures carry a high level of morbidity. This incidence raises the consideration that bone imaging should be included more frequently in the clinical workflow of patients with advanced MPM. Furthermore, complaints of bone pain should prompt early radiographic assessment and if confirmed, appropriate management instigated.

There was no correlation found between presence of bone, visceral or peritoneal/omental metastases and overall survival in this subgroup. This is important prognostic information to inform discussions with newly diagnosed MPM patient. We report on a distinct subgroup of advanced MPM patients. The median overall survival of this reported subgroup was 23.8 months which is significantly longer than published traditional survival statistics for patients with advanced MPM [14, 15]. Over the coming years, we expect treatment options to expand for MPM and for prognosis to improve for the MPM population as a whole, thus the reported cohort may be reflective of future populations of such patients.

Limitations of this study include the retrospective nature of this analysis and the possibility of some metastatic sites not identified on the imaging platforms used. For instance, neurological symptoms prompted brain imaging in this cohort. Thus, asymptomatic and incidental intracerebral metastases may have been undiagnosed. Rates of brain metastases in this study are similar to a previous report [5]. Imaging platforms also changed throughout the course of this review, with plain radiography chosen more frequently a decade ago compared to the current practice of CT thorax preference. Advances in imaging modalities also through the years allowed identification of additional metastatic sites, that may have gone unrecognized a decade prior. As predominantly only one set of images were received from the referring centre, it is not possible to comment upon the timing of the development of metastases in the course of the disease.

## Conclusions

We present a large cohort of mesothelioma patients and detail the incidence of metastatic disease. With 67% of patients diagnosed with metastatic disease advanced MPM should no longer be considered a local disease without the propensity to disseminate and metastasise. In addition, with an almost 20% bone metastases rate, consideration should be given for incorporating routine bone imaging into the cancer care algorithms or symptom-driven bone imaging. No correlation of survival with presence of metastatic disease was seen.

## Data Availability

The datasets used and/or analysed during the current study are available from the corresponding author on reasonable request.

## Abbreviations

CT: Computed tomography
HR: Hazard ratio
MPM: Malignant pleural mesothelioma
MRI: Magnetic resonance imaging
MVP: Mitomycin-C, vinblastine and cisplatin
OS: Overall survival
PET: Positive electron tomography
TACE: Mitomycin-C, gemcitabine and cisplatin
US: Ultrasound
