# Correction to: Radiological evaluation of malignant pleural mesothelioma - defining distant metastatic disease

**DOI:** 10.1186/s12885-021-07999-y

**Published:** 2021-03-08

**Authors:** Dearbhaile Catherine Collins, Raghav Sundar, Anastasia Constantinidou, David Dolling, Timothy Anthony Yap, Sanjay Popat, Mary E. O’Brien, Udai Banerji, Johann Sebastian de Bono, Juanita Suzanne Lopez, Nina Tunariu, Anna Minchom

**Affiliations:** 1 Drug Development Unit, Royal Marsden Hospital/ Institute of Cancer Research, Down Rd., Sutton, SM2 5PT UK; 2 Lung Unit, Royal Marsden Hospital, Fulham Rd., London, SW3 6JJ UK; 3 Lung Unit, Royal Marsden Hospital, Sutton, SM2 5PT UK

**Correction to: BMC Cancer 20, 1210 (2020)**

**https://doi.org/10.1186/s12885-020-07662-y**

Following publication of the original article [1], the authors identified an error in the author name of Anastasia Constantinidou.

The incorrect author name is: Anastasia Constantidinou

The correct author name is: Anastasia Constantinidou

The author group has been updated above and the original article [1] has been corrected.

## References

[1] Collins DC, Sundar R, Constantinidou A (2020). Radiological evaluation of malignant pleural mesothelioma - defining distant metastatic disease. BMC Cancer.

